# Immune Checkpoint Inhibitor-Associated Thrombotic Thrombocytopenic Purpura in a Patient With Metastatic Non-Small-Cell Lung Cancer

**DOI:** 10.7759/cureus.16035

**Published:** 2021-06-29

**Authors:** Samantha De Filippis, Colton Moore, Kristin Ezell, Kunal Aggarwal, Amar H Kelkar

**Affiliations:** 1 Medical Education, University of Medicine and Health Sciences, Camps, KNA; 2 Medical Education, Saint George's University School of Medicine, True Blue, GRD; 3 Medical Education, Ross University School of Medicine, Miramar, USA; 4 Division of Hematology and Oncology, University of Florida College of Medicine, Gainesville, USA

**Keywords:** immune checkpoint inhibitors, thrombotic thrombocytopenic purpura, thrombotic microangiopathy, pembrolizumab, immune related adverse events

## Abstract

Background: Immune-related adverse events (irAEs) are secondary reactions related to treatment with immune checkpoint inhibitors (ICIs). There have been six cases published reporting on an association between patients undergoing treatment with ICIs and the occurrence of acquired thrombotic thrombocytopenic purpura (TTP).

Case report: We report a 61-year-old male receiving treatment with chemoimmunotherapy followed by pembrolizumab maintenance therapy for advanced non-small-cell lung cancer, presenting with bleeding symptoms, anemia, and thrombocytopenia. The patient received pembrolizumab seven times in total, in three-week cycles. Laboratory testing demonstrated hemolytic anemia, which, in combination with other findings, suggested thrombotic microangiopathy (TMA). PLASMIC scoring and specialized testing with ADAMTS13 activity and inhibitor confirmed a diagnosis of TTP. The patient was started on therapy with plasmapheresis and glucocorticoids, resulting in clinical improvement. The patient chose to leave the hospital under the care of home hospice and died approximately one month after being discharged.

Conclusions: Of the six cases of ICI-induced TTP, only one other was treated with pembrolizumab to our knowledge to date. Our patient experienced an adverse reaction marked by thrombocytopenia and hematuria after drug exposure. With symptom improvement after ICI discontinuation and recurrence on readministration, a presumptive diagnosis of ICI-associated TTP was made. This case report and literature review emphasize the need for close observation of patients undergoing ICI therapy for potential rare irAEs. The further investigation aimed at the study of risk factors, disease severity, and treatment response to this form of secondary TTP is needed to guide treatment decisions.

## Introduction

Lung cancer is the leading cause of cancer deaths in the United States, with non-small-cell lung cancer (NSCLC) making up the vast majority (85%) of diagnoses. Screening and treatment for NSCLC have been evolving rapidly over the last decade, and in recent years, immune checkpoint inhibitor (ICI) therapy has come to the forefront as a first-line treatment [1]. ICIs are monoclonal antibodies that work by inhibiting pathways that maintain self-tolerance that have been overexpressed on tumor cells, or in the tumor microenvironment, to escape immune surveillance, primarily by T cells. Most ICIs currently in use inhibit the programmed death 1 (PD-1) and programmed death-ligand 1 (PD-L1) pathway, including pembrolizumab, an anti-PD-1 monoclonal antibody. In this pathway, the PD-1 receptor on the cell surfaces of tumor-infiltrating lymphocytes, particularly regulatory T cells, binds to PD-L1 on the surface of host cells resulting in immune tolerance [1]. Due to upregulation of PD-L1 on tumor cell surfaces, pembrolizumab was first approved by the U.S. Food and Drug Administration for treatment of advanced NSCLC in October 2015, with subsequent approvals for several other cancer types including bladder, cervical, gastroesophageal junction, head and neck, hepatocellular, Hodgkin lymphoma, Merkel cell, primary mediastinal B cell lymphoma, and stomach, as well as microsatellite instability-high or deficient mismatch repair metastatic solid tumors [2]. It is also the investigative focus of many ongoing clinical trials. Nivolumab, like pembrolizumab, is an anti-PD-1 inhibitor. Ipilimumab, another ICI, is a cytotoxic T lymphocyte-associated protein 4 (CTLA-4) blocker [3].

Although ICIs are increasingly used as a new modality for cancer treatment, they also bring with them new challenges in management including immune-related adverse events (irAEs). The onset of irAEs may be dose- or therapy-dependent, time-delayed, and arise in any organ system. The overall incidence of irAEs with pembrolizumab therapy was reported as 41.0% [4]. It has been demonstrated that combination therapies lead to higher rates of irAEs [4].

Numerous irAEs were reported during and after clinical trials, and it is important for there to be high clinical suspicion for irAEs for there to be timely diagnosis and appropriate management. Common irAEs include skin manifestations such as maculopapular rash, pruritus, and vitiligo; gastrointestinal manifestations such as colitis and hepatitis; endocrine manifestations such as hypothyroidism and hypophysitis; pulmonary manifestations such as pneumonitis; and rheumatic manifestations such as inflammatory arthritis and polymyalgia-like syndromes. Rarely, cardiovascular, renal, neurologic, ophthalmologic, and hematologic toxicities have been described [2,3].

While hematologic toxicities are atypical, they represent serious and under-studied complications of ICI therapy. Case reports describing hematologic irAEs published to date remain varied and sparse. Hemolytic and aplastic anemia, thrombocytopenia, acquired hemophilia A, and lymphopenia are among the most commonly reported hematologic irAEs [2,3]. Herein, we present a case of ICI-associated thrombotic thrombocytopenic purpura (TTP) in a 61-year-old man with advanced NSCLC who was treated with pembrolizumab. Only six other cases of ICI-related TTP have been reported to date [5-10].

## Case presentation

**Figure 1:**
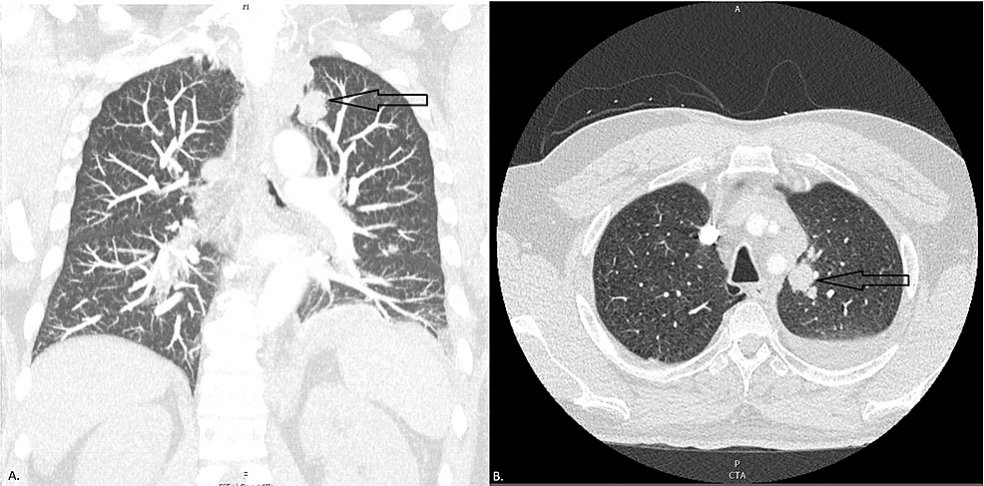
Computed tomography of the thorax with contrast at the time of diagnosis with lung cancer (A) Coronal and (B) axial views demonstrate a large 2.3 × 1.4 cm^2^ nodule in the medial aspect of the left upper lobe suspicious for primary lung cancer (black arrows) with a moderate-sized left pleural effusion and compressive atelectasis in the left lung base.

**Figure 2:**
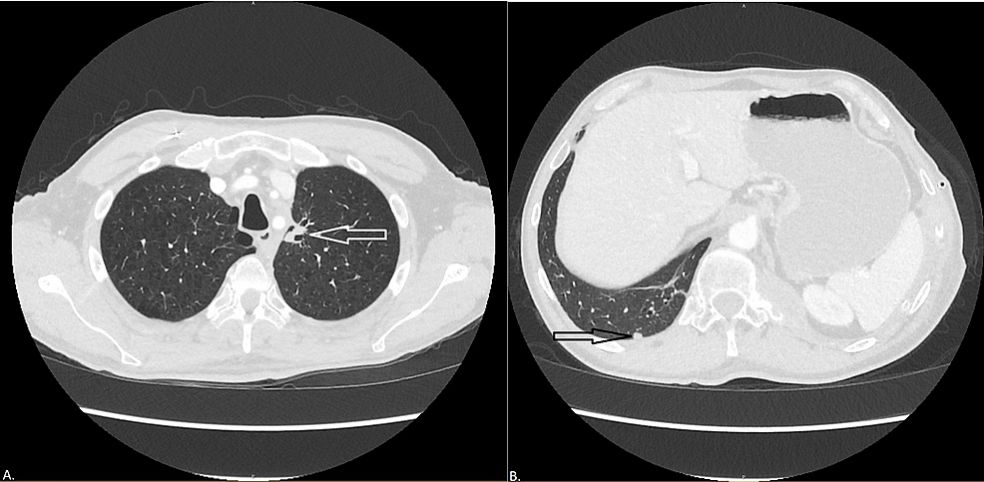
Computed tomography of the thorax with contrast at the time of lung cancer restaging after induction chemoimmunotherapy (A) Axial thoracic view demonstrates reduction of the overall disease burden, with the cavitary left apical nodule now decreased in size and lobulated with peripheral cavitary changes, with the solid portion measuring 1.6 × 0.8 cm^2^ (white arrow). (B) Axial abdominal view demonstrates additional satellite nodules have decreased in size, but there is a new 6 mm subpleural pulmonary nodule in the right lower lobe (black arrow).

**Figure 3:**
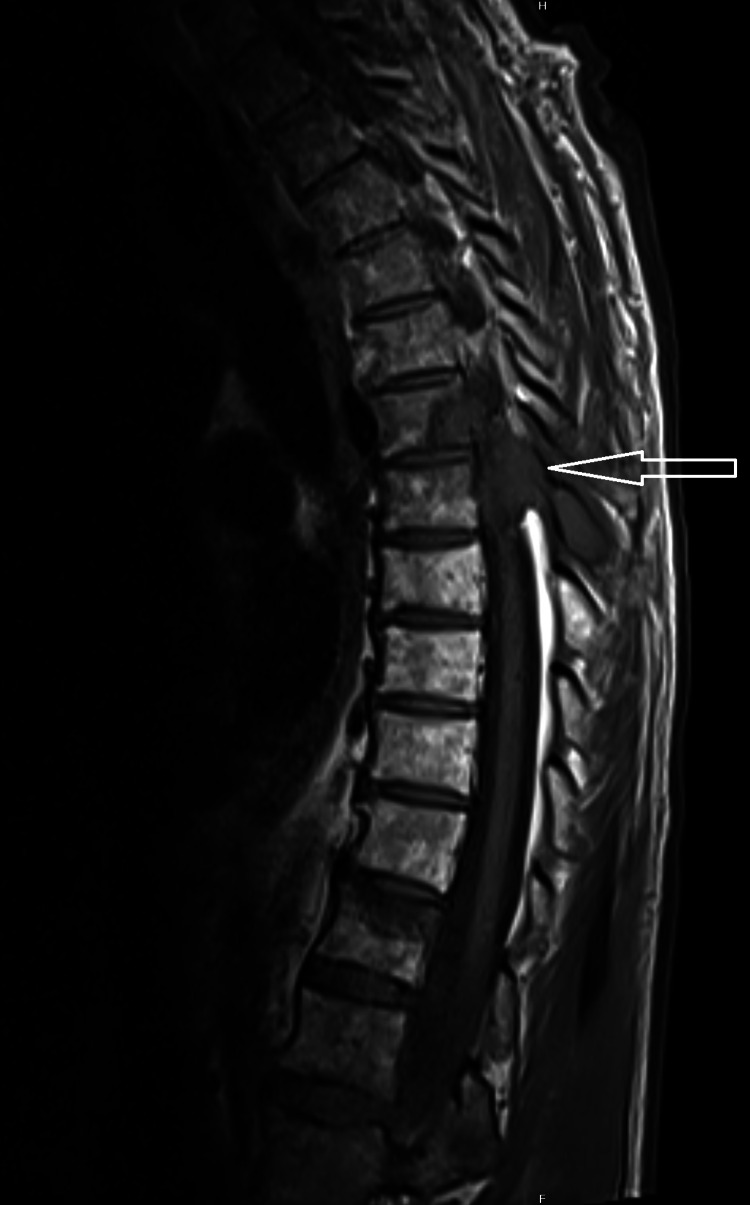
Magnetic resonance imaging of the thoracic spine at the time of lung cancer progression Sagittal view demonstrates a mass at T6-7 extending from the medial left rib into the T6 and T7 vertebral body and extending into the spinal canal with contact and displacement of the spinal cord measuring approximately 7 cm × 4.4 cm greatest dimensions in the axial plane and greatest vertical dimension approximately 4.7 cm (white arrow). Anteriorly this lesion abuts the posterior wall of the thoracic aorta.

The case involves a 61-year-old male with a nine-month history of stage IV (T2aN2M1) NSCLC with mediastinal lymphadenopathy, extensive left pleural involvement, and erosion into the left posterior rib. Lung cancer was originally diagnosed following three months of progressive left lower rib pain, initially attributed to a muscle strain. Due to the increasing intensity of the patient’s pain, he presented to the emergency room, where computed tomography (CT) of the thorax revealed a 3 cm left upper lobe nodule and destruction of the left posterior ninth rib (Figure 1). The patient was referred to medical oncology and radiation oncology and was discharged after fine-needle aspiration of the primary lesion confirmed squamous cell carcinoma. He then underwent 17 fractions of stereotactic body radiation therapy (SBRT) to the left posterior ribs. The patient subsequently developed worsening dyspnea on exertion prior to receiving systemic therapy and was readmitted for left pleural effusion and was eventually discharged after placement of a flexible drainage catheter (PleurX™), with drainage fluid confirming malignant effusion. The patient was then consented and scheduled for induction chemoimmunotherapy with carboplatin, paclitaxel, and pembrolizumab with zoledronic acid infusions to control hypercalcemia [1]. Prior to initiation of therapy, the platelet level was 165,000/µL. The patient completed five cycles of chemoimmunotherapy without any high-grade adverse events and restaging imaging revealed a mixed response with significant tumor burden reduction on CT (Figure 2). The patient was then put on maintenance therapy with pembrolizumab 200 mg every three weeks, but only completed two cycles due to the development of anorexia, unexplained weight loss of 10 lbs, and diminishing function in activities of daily living. After an extended discussion on his goals of care, the patient elected to discontinue immunotherapy and manage with surveillance imaging. However, he was subsequently found to have a T6-7 growing mass on MRI (Figure 3) that was managed with five fractions of SBRT.

**Figure 4:**
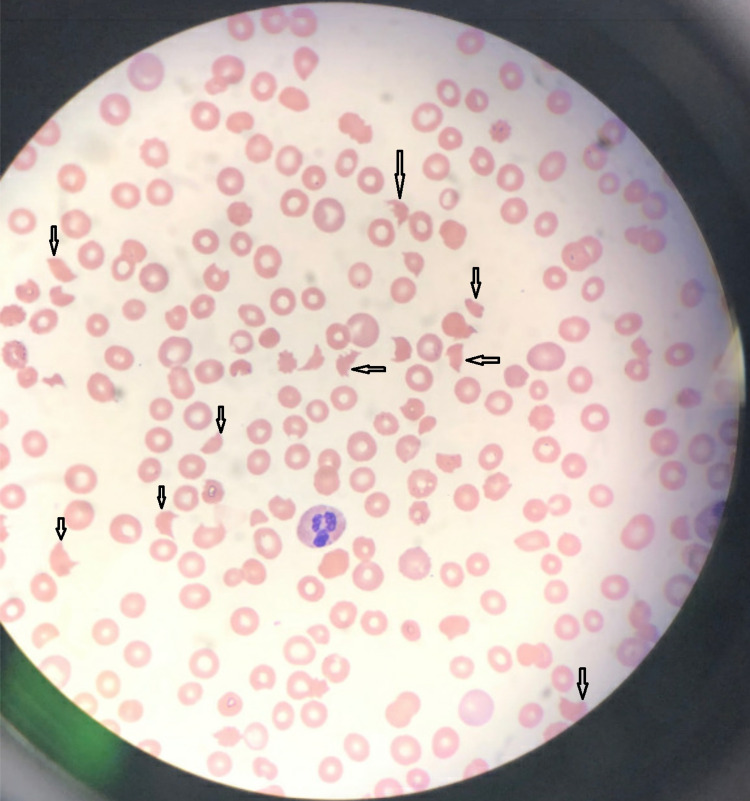
Peripheral blood smear at the time of presentation with thrombotic thrombocytopenic purpura Peripheral blood smear revealed near absent platelets, without evidence of clumping, and many schistocytes (black arrows), with >8 per high-powered field.

**Figure 5:**
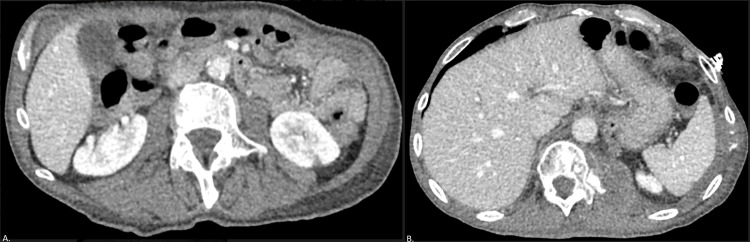
Computer tomography of the abdomen and pelvis with contrast at the time of presentation with thrombotic thrombocytopenic purpura (A) Axial upper- and (B) mid-abdominal views demonstrated no evidence of hepatosplenomegaly or retroperitoneal hemorrhage. No evidence of biliary ductal dilatation or focal liver lesions and the portal vein is patent. The spleen, pancreas, and kidneys are normal. There is cholelithiasis without evidence of acute cholecystitis. The bladder demonstrates distention without pelvic mass. There is no evidence of bowel obstruction or pneumoperitoneum. There are no ascites or evidence of retroperitoneal hemorrhage.

Approximately two weeks after completing SBRT, and approximately five weeks after his last dose of pembrolizumab, the patient presented to the emergency department with gross hematuria, acute on chronic thrombocytopenia, and acute on chronic normocytic anemia. On physical examination, presenting vital signs were stable without fever and there was no bruising or neurological dysfunction; however, jaundice, scleral icterus, and costovertebral tenderness were noted. The patient’s only other medications were daily ferrous sulfate and a daily multivitamin supplement. In the emergency department, the hematology service was urgently consulted and initial labs revealed normal serum creatinine, blood urea nitrogen, international normalized ratio (INR), activated partial thromboplastin time (aPTT), and fibrinogen, with a low platelet count of 8,000/µL, low hemoglobin of 7.2 g/dL, high mean corpuscular volume (MCV) of 98.5 fL, high corrected reticulocyte count of 5.0%, high lactose dehydrogenase (LDH) of 615 IU/dL, low haptoglobin of <30 mg/dL, and high total and indirect bilirubin of 4.2 mg/dL and 3.3 mg/dL, respectively (Table 1). Peripheral blood smear revealed near absent platelets, without evidence of clumping, and many schistocytes, with >8 per high-powered field (Figure 4). Imaging by CT of the abdomen and pelvis showed no evidence of hepatosplenomegaly or retroperitoneal hemorrhage and did not visualize the primary tumor site (Figure 5). Based on these findings, a PLASMIC score was calculated to be 5 points out of 7, indicating intermediate risk for TTP. Due to access at our facility, on the date of admission, we were able to confirm the severe ADAMTS13 deficiency and diagnosis of TTP with an ADAMTS13 enzyme activity of 10-12%.

**Table 1:** Laboratory findings during hospitalization for thrombotic thrombocytopenic purpura TPE: plasmapheresis. ^Taken one day prior to completion of TPE.

Test	Measured value prior to TPE initiation	Measured value following TPE completion	Reference values	Units
Lactate dehydrogenase	615	179	120–250	U/L
Reticulocyte count	5	3.5	0.6–2.6	%
Hematocrit	16.2	23.9	38.5–50.0	%
Hemoglobin	5.5	8	13.2–17.1	g/dL
Haptoglobin	30	86	43–212	mg/dL
Platelet count	7	82	150–450	10^9^/L
ADAMTS13 activity	12	45^	68–163	%
ADAMTS13 inhibitor	70	--	<0.4	BEU
Bilirubin, indirect	2	0.4^	0.2–1.2	mg/dL
Prothrombin time/international normalized ratio	14.9/1.9	--	9–11.5/0.9–1.1	sec
Partial thromboplastin time	26	26	23–32	sec
Fibrinogen	422	253	175–425	mg/dL

**Figure 6:**
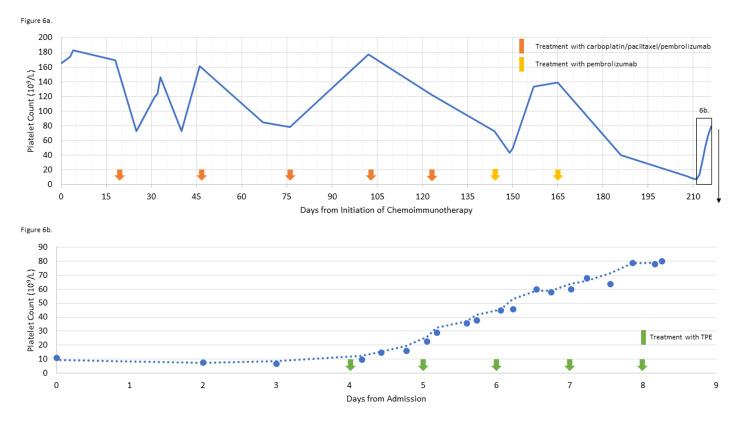
Platelet counts from the initial presentation with thrombotic thrombocytopenic purpura Yellow and orange arrows indicate chemoimmunotherapy cycle start dates. Green arrows represent dates receiving plasmapheresis.

**Figure 7:**
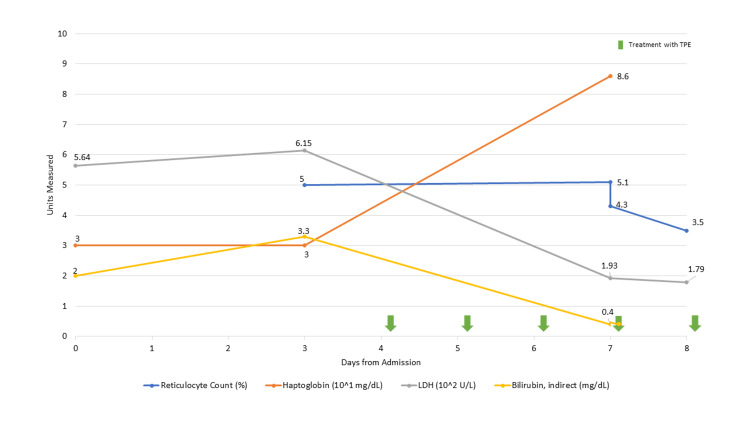
Bilirubin, lactate dehydrogenase, haptoglobin, and reticulocyte count during hospitalization for thrombotic thrombocytopenic purpura Green arrows represent dates receiving plasmapheresis.

Following the diagnosis of TTP, the patient was admitted to the oncology service and emergent plasmapheresis (TPE) was initiated overnight. After the first TPE treatment, the patient became hypotensive and his anemia worsened to a hemoglobin level of 5.5 g/dL; therefore, he was transferred to the intensive care unit. At this point, additional testing revealed an ADAMTS13 inhibitor level of 70% and an antibody titer of 1.8 Bethesda units. He received TPE for a total of five consecutive days with concurrent intravenous methylprednisolone for three days followed by daily oral prednisone of 1 mg/kg. He remained hemodynamically stable throughout the remainder of the treatment course. The patient's platelet count significantly recovered to 82,000/µL following treatment (Table 1; Figures 6 and 7). However, he ultimately chose to leave the hospital under the care of home hospice due to further decline in function. He did not receive further treatment for TTP or NSCLC, except for the continuation of prednisone to delay TTP relapse for as long as possible. The patient died at home approximately one month after being discharged.

## Discussion

Thrombotic microangiopathy (TMA) is defined as microangiopathic hemolytic anemia (MAHA) with thrombocytopenia and end-organ damage due to the formation of microvascular thrombi. TMA subtypes include disseminated intravascular coagulation, atypical hemolytic uremic syndrome, cancer-related TMA (CR-TMA), and TTP [11,12]. In this case, a 61-year-old male receiving treatment with chemoimmunotherapy followed by pembrolizumab maintenance therapy for advanced NSCLC with metastasis to the rib and spine presented with bleeding symptoms, anemia, and thrombocytopenia. The patient’s most recent medical care prior to his hospitalization included SBRT and pembrolizumab exposure two and five weeks prior to presentation, respectively. Thus, the presence of NSCLC and proximity of recent ICI therapy, as well as the lack of exposure to other potential causes, raised concern for both ICI-related TTP and CR-TMA.

Thrombotic thrombocytopenic purpura is often diagnosed by severe thrombocytopenia, reticulocytosis, elevated LDH levels, elevated indirect bilirubin, low or absent haptoglobin levels, and very low ADAMTS13 activity [13]. One validated scoring tool to aid in clinical judgment, particularly at sites with available ADAMTS13 testing, is the PLASMIC score, which gives a point for each of the following: platelet count <30 × 109/L, hemolysis, no active cancer within the past year, no history of solid organ transplant (SOT) or blood and marrow stem cell transplant (BMT), MCV < 90 fL, INR < 1.5, and serum creatinine < 2.0 mg/dL [11]. Therefore, PLASMIC scoring and specialized testing with ADAMTS13 activity and inhibitor can be used to differentiate a diagnosis of TTP, from CR-TMA clinically.

Thrombotic thrombocytopenic purpura can be hereditary or acquired, with most cases being acquired. Acquired TTP (aTTP) is caused by an autoantibody forming against ADAMTS13, which can be secondary to bacterial infections, autoimmune diseases such as systemic lupus erythematous, pregnancy, drugs (e.g., mitomycin C, cyclosporine, quinine, clopidogrel, ticlopidine, and hormone therapies), HIV infection, pancreatitis, active cancer, surgery, and transplantation including blood and marrow stem cell transplant as well as a solid organ transplant. However, approximately 50% of cases have been reported to be idiopathic [13].

Given the immune-related mechanism of ICI adverse effects, it could be inferred that an autoimmune reaction such as aTTP was possible. In attributing the cause of an adverse effect to a drug, one tool for defining probability is the Naranjo Adverse Drug Reaction Probability Scale [14]. Our patient scored points for prior reported cases (+1), presence of the adverse event after drug exposure (+2), adverse event improvement after antagonist administration (+1), and a lack of possible alternative causes due to ruling out CR-TMA (+2), giving him a score of 6 out of 9, which correlated to a probable reaction. This supports the presumptive diagnosis of ICI-associated TTP.

**Figure 8:**
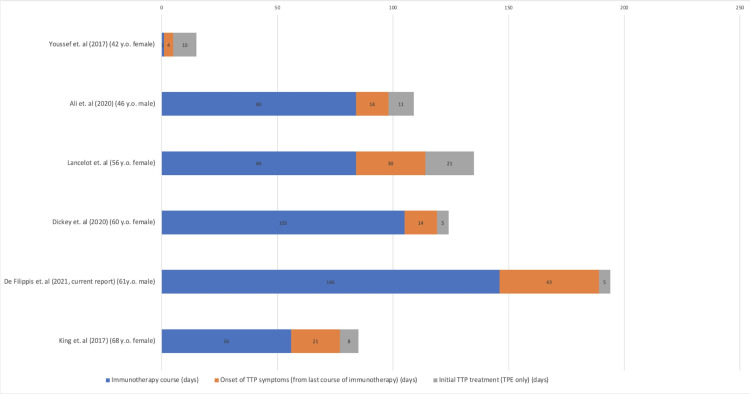
Literature review treatment summary Literature review summarizing the duration of treatment across five other reported cases of ICI-induced TTP. Cases included in this figure: Youssef et al. [6], Ali et al. [8], Lancelot et al. [9], Dickey et al. [5], De Filippis et al. (current report), King et al. [10], respectively. N.B. The duration of immunotherapy could not be clearly determined definitively in Lancelot et al., but the case was included in this figure to depict the onset of TTP and duration of TPE. LaFranchi et al. were excluded from this figure due to the lack of a clearly defined duration of events. Ali et al. did not have 11 consecutive days of TPE, he was treated for 6 days, platelets recovered so the treatment was paused and 5 days later platelets depleted so he was started on TPE for another 5 days.

In total, six cases of ICI-associated TTP have been reported (Table 2) [5-10]. There was only one other case of pembrolizumab-associated TTP; however, we did find an additional case of pembrolizumab-associated TMA [5,15]. An additional four cases were reported of TTP related to combination therapy with ipilimumab and nivolumab, and one case was reported with ipilimumab monotherapy (Table 2) [6-10]. Of the seven total cases (including our case), the median age is 60 years old with a range of 28 years. The median number of days between cessation of immunotherapy and onset of TTP symptoms was 17 days, though Lafranchi et al. were excluded due to lack of precise immunotherapy dates (Figure 8). There were four female patients out of seven, and a 50% reported survival rate after therapy. One patient decided not to pursue treatment. In every case, the diagnosis of TTP was confirmed by severe ADAMTS13 deficiency and the presence of elevated ADAMTS13 enzyme inhibitors.

**Table 2:** Literature review of cases of immune checkpoint inhibitor-associated thrombotic thrombocytopenic purpura DM, diabetes; HTN, hypertension; HLD, hyperlipidemia; COPD, chronic obstructive pulmonary disease; CAP, community-acquired pneumonia; ARF, acute renal failure; IVIg, intravenous immunoglobulin; pRBC, packed red blood cell transfusion.

Author (Year)	De Filippis et al. (current report)	Dickey et al. [5]	Youssef et al. [6]	LaFranchi et al. [7]	Ali et al. [8]	Lancelot et al. [9]	King et al. [10]
Patient description	61-year-old male	60-year-old female	42-year-old female	70-year-old male	46-year-old male	56-year-old female	68-year-old female
Underlying malignancy	Stage IV (T2aN2M1) non-small-cell lung cancer with rib and spine metastases complicated by hypercalcemia	Stage IV non-small-cell lung cancer with brain metastases (75% PD-1 expression)	Stage IV renal cell carcinoma with extensive sarcomatoid features	Melanoma with brain metastases	Stage IV renal cell carcinoma of the left kidney with pulmonary and cutaneous metastases	Stage IV metastatic melanoma	Stage III (pT3N2M0) high-risk, ulcerated, spindle cell melanoma
Medical history and concurrent issues	CAP	DM, HTN, HLD, COPD, CAP	None reported	None reported	None reported	None reported	Otitis externa, ARF
Immune checkpoint inhibitor regimen	Pembrolizumab* × 7 cycles, every 3 weeks. *First 5 cycles in combination with carboplatin and paclitaxel	Pembrolizumab × 5 cycles, every 3 weeks	Nivolumab and Ipilimumab × 1 cycle	Nivolumab and Ipilimumab	Nivolumab and Ipilimumab × 4 cycles, every 3 weeks	Nivolumab and Ipilimumab × 4 cycles, every 3 weeks	Ipilimumab × 4 cycles, every 3 weeks
Time from the last dose to presentation with TTP	46 days	14 days	4 days	18 days	14 days	30 days	19 days
TTP treatment	TPE, methylprednisolone, prednisone	TPE, methylprednisolone, prednisone	TPE, methylprednisolone, rituximab	No TPE	TPE, prednisone, rituximab, caplacizumab	TPE, prednisone, rituximab	TPE, methylprednisolone, rituximab, IVIg, pRBC
Outcome	Unclear if complete response achieved; died off a therapy on hospice	Unclear response; died after therapy	Complete response	Died without receiving therapy (patient choice)	Durable remission	Unclear response; multiple relapses; died after therapy	Durable remission

Frontline therapy for aTTP in the acute setting involves urgent total plasma exchange (TPE) repeated over multiple sessions combined with glucocorticoids, often starting with intravenous methylprednisolone before transitioning to oral prednisone. Drug-induced TTP is managed by immediate cessation of the causative agent along with the standard of care therapy for aTTP [11]. However, in the case of ICIs, which tend to have delayed onset of effect and irAEs, it is unclear how long the effect could continue after cessation of the drug. In 2019, caplacizumab, a humanized, bivalent, variable-domain-only immunoglobulin fragment, was approved in the United States for use in the first-line setting. However, recent analysis has demonstrated that caplacizumab added to the standard of care is not cost-effective [16]. Our patient was not started on caplacizumab due to not meeting criteria based on hospital protocols to add the agent in situations of cardiac or neurologic complications of TTP, as well as an adequate response to therapy after completion of at least four sessions of TPE.

The goal of therapy for an acute episode of aTTP is to increase platelet counts, relieve ischemia, and eradicate the autoantibody. Complete response is defined as a platelet count greater than 150 × 103/µL for at least two consecutive days with normalizing LDH and clinical recovery. A durable remission is defined as a complete response with continued normal platelet counts for at least 30 days after discontinuation of TPE and normalization of platelets [13].

## Conclusions

Immune checkpoint inhibitor therapies represent the first step in the ongoing paradigm shift of cancer treatment moving away from highly toxic chemotherapy and towards targeted therapies. However, as ICI therapy becomes more prominent, it will be imperative for all physicians to be able to recognize and manage the irAEs unique to ICI therapy. Our case report and literature review add to the limited literature describing the characteristics, diagnosis, and management of patients with ICI-associated TTP and demonstrate the need for further study of the risk factors, onset, severity, and response to therapy for rarer ICI irAEs.
